# Static and Dynamic Accuracy of an Innovative Miniaturized Wearable Platform for Short Range Distance Measurements for Human Movement Applications

**DOI:** 10.3390/s17071492

**Published:** 2017-06-24

**Authors:** Stefano Bertuletti, Andrea Cereatti, Daniele Comotti, Michele Caldara, Ugo Della Croce

**Affiliations:** 1 Information Engineering Unit, Department of Information Engineering, Political Sciences and Communication Sciences, University of Sassari, Sassari 07100 (SS), Italy; acereatti@uniss.it (A.C.); dellacro@uniss.it (U.D.C.); 2 Department of Electronics and Telecommunications, Politecnico di Torino, Torino 10129 (TO), Italy; 3 Department of Engineering and Applied Sciences, University of Bergamo, Dalmine 24044 (BG), Italy; daniele.comotti@unibg.it (D.C.); michele.caldara@unibg.it (M.C.)

**Keywords:** wearable devices, inertial sensors, time-of-flight, proximity sensors, distance estimation, human movement analysis, step width

## Abstract

Magneto-inertial measurement units (MIMU) are a suitable solution to assess human motor performance both indoors and outdoors. However, relevant quantities such as step width and base of support, which play an important role in gait stability, cannot be directly measured using MIMU alone. To overcome this limitation, we developed a wearable platform specifically designed for human movement analysis applications, which integrates a MIMU and an Infrared Time-of-Flight proximity sensor (IR-ToF), allowing for the estimate of inter-object distance. We proposed a thorough testing protocol for evaluating the IR-ToF sensor performances under experimental conditions resembling those encountered during gait. In particular, we tested the sensor performance for different (i) target colors; (ii) sensor-target distances (up to 200 mm) and (iii) sensor-target angles of incidence (AoI) (up to $60^{\circ }$). Both static and dynamic conditions were analyzed. A pendulum, simulating the oscillation of a human leg, was used to generate highly repeatable oscillations with a maximum angular velocity of 6 rad/s. Results showed that the IR-ToF proximity sensor was not sensitive to variations of both distance and target color (except for black). Conversely, a relationship between error magnitude and AoI values was found. For AoI equal to $0^{\circ }$, the IR-ToF sensor performed equally well both in static and dynamic acquisitions with a distance mean absolute error <1.5 mm. Errors increased up to 3.6 mm (static) and 11.9 mm (dynamic) for AoI equal to $\pm 30^{\circ }$, and up to 7.8 mm (static) and 25.6 mm (dynamic) for AoI equal to $\pm 60^{\circ }$. In addition, the wearable platform was used during a preliminary experiment for the estimation of the inter-foot distance on a single healthy subject while walking. In conclusion, the combination of magneto-inertial unit and IR-ToF technology represents a valuable alternative solution in terms of accuracy, sampling frequency, dimension and power consumption, compared to existing technologies.

## 1. Introduction

Dynamic stability is essential to efficiently and safely perform motor tasks such as gait or to maintain the human body in a stable upright posture [1]. A common and simple strategy to increase stability consists of widening the base of support. Excessive variability of the step width (distance between feet when they are both in contact with the ground) is also associated with gait instability [2], risk or fear of falling [3,4,5] and with the severity of movement disorders [6]. Traditionally, step width is determined in the laboratory setting using either an electronic walkway or a stereo-photogrammetric system [7,8]. These solutions provide accurate estimates, but their use is limited to the laboratory environment and they are quite expensive. Nowadays, the use of low cost wearable technology for objective and ecological assessment of gait, stability and balance during daily life is growing [9,10,11,12,13,14]. However, this technology does not allow for direct measurements of the relative position of body segments such as the feet, which are necessary to estimate the base of support or step width variation over time [15]. Some researchers have combined inertial measurement unit (IMU) with ultrasounds (US), light intensity infrared (IR-LI) and Video camera (VC) sensors [16,17,18,19]. US sensors estimate a distance by measuring the time required by an ultrasonic wave to travel from the transmitter to the receiver [16,20]. IR-LI sensors determine the distance by measuring the amount of light reflected from an object [17,18,21]. VCs have been used to measure the distance from a matrix of infrared LEDs positioned in front of it [19,22]. The US technology is characterized by an output data rate (*ODR*) up to 40 Hz and a power consumption up to 15–30 mA, which may limit the use of this technology for long-term monitoring applications [16,20]. IR-LI sensor technology allows for a higher *ODR* (∼60 Hz), but its accuracy is heavily dependent on experimental factors, such as color and reflectance of the target surface [23]. A main drawback of both US and VC technologies is that two distinct modules, a transmitter and a receiver, must be positioned on the points of interest. Furthermore, VC sensors employed in a previous study [19] were bulky (e.g., Pointgrey *Firefly MV*: 44×34×32 mm${}^{3}$ [22]) and therefore they could impede the subject movement. Another relatively recent solution for distance estimation is the use of Infrared Time-of-Flight proximity sensors (IR-ToF). This technology provides an estimate of the distance between the sensor and the target based on the time that an electromagnetic wave takes to travel a distance (Time-of-Flight) or, more correctly, by measuring the phase shift between the emitted and the reflected signals [24]. The main advantages offered by this technology are: (i) transmitter and receiver integrated in the same module; (ii) small sensor dimensions; (iii) accuracy independent from experimental factors (e.g., intensity of the ambient light); (iv) high *ODR* (up to 50 Hz) and (v) low power consumption (∼2–5 mA). Thus, IR-ToF proximity sensors appear to be suitable for use for human movement applications.

A preliminary study using an evaluation kit board revealed that IR-ToF sensors (*VL6180X*, STMicroelectronics, Geneva, Switzerland) can provide a better accuracy compared to alternative technologies [25]. However, in another preliminary study using the same sensor, it was suggested that variations of the angle of incidence (AoI) of the emitted infrared ray could heavily affect the IR-ToF performance when analyzing inter-foot distance (IFD) during gait [26]. Furthermore, it should be noticed that, in general, the sensor manufacturers (e.g., STMicroelectronics) only report in the datasheet the sensor performance under very specific and controlled conditions (e.g., in static conditions, for a given target color, at fixed *ODR*, etc.) [27]. Therefore, for a proper use of an IR-ToF proximity sensor in combination with inertial sensing unit for human movement applications, it is crucial to evaluate the system performance under working conditions simulating the real scenarios.

Few previous studies have proposed to integrate IMU data with distance measurements, based on the abovementioned technologies, for analyzing human gait. Arami et al. [17] presented a wearable system for the estimation of the foot clearance during gait. Different system configurations, from one to three IR-LI sensors, were investigated on a single healthy subject. Trojaniello et al. [18] combined an IR-LI sensor with an IMU and performed, on a single subject, Inter-Foot Distance (IFD) estimates in both static and dynamic conditions (leg swinging and walking). Weenk et al. [16] used an extended Kalman filter to fuse ultrasound range estimates and inertial sensor data for the estimation of the step length and stride width on three subjects. Hung et al. [19] fused data recorded using a VC to compensate the inter-shoe position estimate provided by an inertial navigation algorithm. Data were recorded on one subject while walking. Finally, Duong et al. [28] presented an algorithm for the estimation of a single foot pose by fusing data provided by two IR-ToF sensors and an IMU during different motor tasks (walking, dancing steps, jumping and kicking a ball) performed by a single subject. Unfortunately, none of the abovementioned studies, with the exception of [18], reported the errors associated with the distance estimated by the specific proximity sensor employed under static and dynamic conditions.

In this paper, we developed a platform, the D-MuSe (Distance-MultiSensing), integrating a state-of-the-art magnetic and inertial measurement unit (MIMU) with an IR-ToF proximity sensor. We proposed a thorough evaluation protocol for testing its performance in static conditions for different target colors and in both static and dynamic conditions, similar to those encountered in human movement, by varying the target distance and AoI. In addition, an example of the use of the D-MuSe platform for the estimation of the IFD during gait on a single healthy subject was given.

## 2. Materials and Methods

### 2.1. Hardware Description

**Figure 1:**
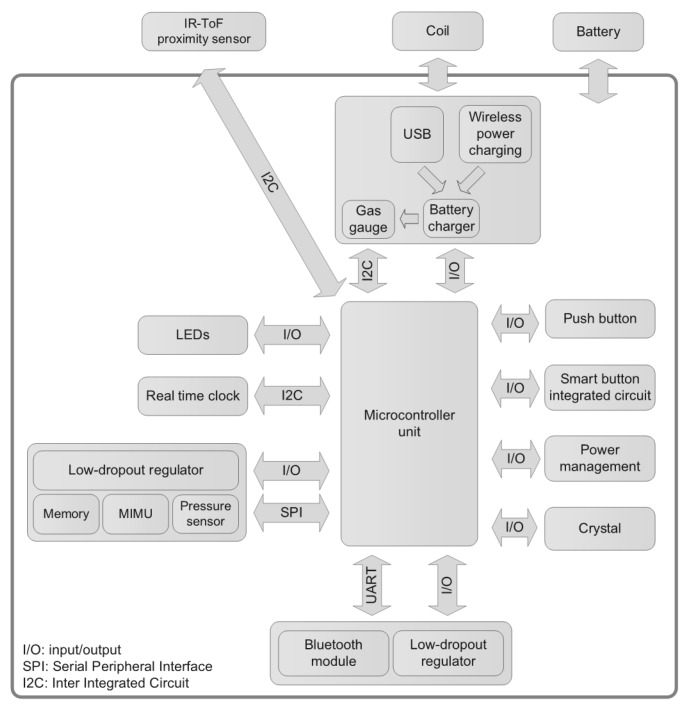
Block diagram of the D-MuSe. The system consists of an ultra-low-power core (*STM32F4*) and low-power sensors with an advanced power management architecture. The radio frequency communication is provided by a Bluetooth module (*BT33* provided by Amp’ed RF/STMicroelectronics, San Jose, CA, USA [29]).

The D-MuSe platform, which includes an MIMU and an IR-ToF proximity sensor was developed. The specific advanced design aims at providing a wireless low-power system with high processing capabilities and a small form factor. As shown in Figure 1, the platform is augmented with additional sensing units for a variety of potential applications.

#### 2.1.1. Microcontroller

The processing core (*STM32F411*) is an ultra-low-power 32-bit controller (7×7 mm${}^{2}$) [30] with 125 DMIPS (Dhrystone million instructions per second) peak capability and an extremely low-power consumption scalable down to 100 μA/MHz (typical consumption in run mode).

#### 2.1.2. Geomagnetic and Inertial Module

The D-MuSe platform integrates in a single chip a 9-axis magnetic and inertial measurement unit (*LSM9DS1*) [31]. The *LSM9DS1* includes a 3D accelerometer (up to ±16 g), a 3D gyroscope (up to ±2000 ${}^{\circ }$/s) and a 3D Magnetometer (up to ±16 Gauss) in a 3.5×3 mm${}^{2}$ package. For this specific study, magneto-inertial data were sampled at 100 Hz and the full scales were set to ±4 g, ±500 ${}^{\circ }$/s and ±4 Gauss for the accelerometer, gyroscope and magnetometer, respectively.

#### 2.1.3. IR-ToF Proximity Sensor

**Figure 2:**
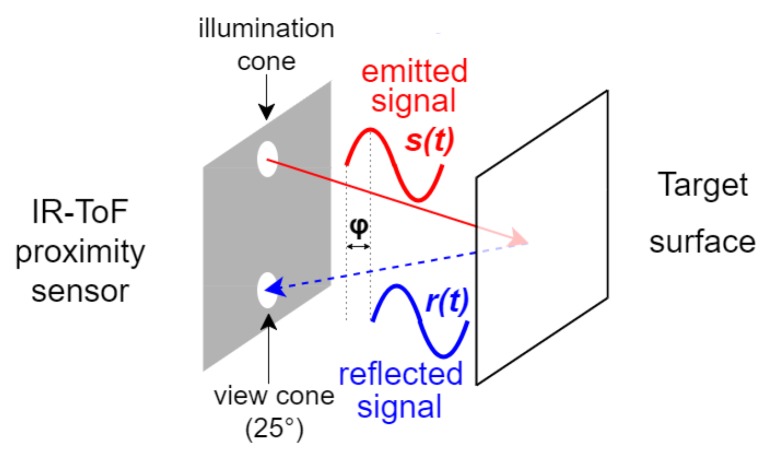
The Infrared Time-of-Flight proximity sensor (IR-ToF) provides the distance estimate from the target reflecting surface by measuring the phase shift φ between the emitted *s(t)* and the reflected *r(t)* signals.

The IR-ToF proximity sensor (*VL6180X*) [27] provides proximity estimates in the range of 0–600 mm. The distance is estimated by measuring the phase shift φ between the radiated *s(t)* and the reflected *r(t)* IR waves (Figure 2):

(1) $$s\left(t\right)=\sin\left(2\pi f_{m}t\right),$$

(2) $$r\left(t\right)=R\cdot \sin\left(2\pi f_{m}t-\varphi \right)=R\cdot \sin\left[2\pi f_{m}\left(t-\frac{2d}{c}\right)\right],$$

where *R* is a reflection coefficient, *c* is the speed of light ($3\times 10^{8}$ m/s) and $f_{m}$ is the modulation frequency of the radiated and reflected signals. Once φ is measured (e.g., phase comparator circuit), the distance *d* between the position of the IR emitter and the target can be calculated from Equation (2) as follows:

(3) $$d=\frac{c}{4\pi f_{m}}\cdot \varphi .$$

**Figure 3:**
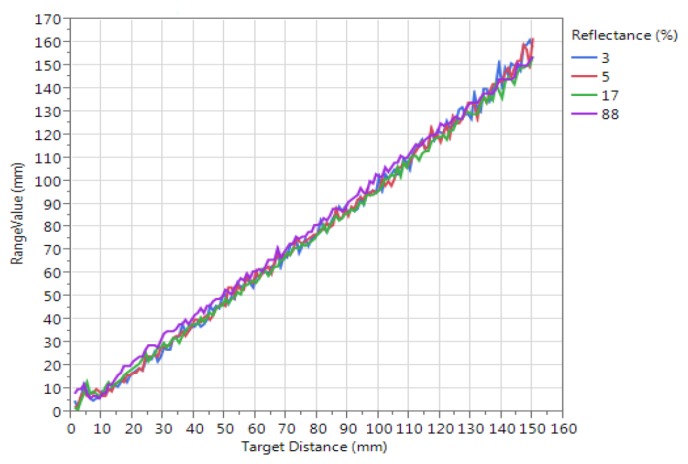
Typical ranging performance of the *VL6180X* proximity sensor, provided by STMicroelectronics (Geneva, Switzerland) [27], for different target reflectance (3%, 5%, 17% and 88%) by varying the range from 0 to 150 mm.

The accuracy in static conditions in the range of 0–150 mm, reported in the *VL6180X* datasheet [27], is shown for different target reflectances in Figure 3.

For this specific study, the IR-ToF proximity sensor sampling rate was set to 50 Hz (maximum frequency allowed), and, since the maximum distance between feet during gait is generally less than 200 mm [3,5,32], the measurement range was set to 0–200 mm. To improve the IR-ToF accuracy, an ad hoc calibration between 0 and 200 mm was performed. The firmware of the platform was programmed to return a “0” value when no light reflection was observed.

#### 2.1.4. Connectivity

The D-MuSe supports both wired and wireless communication: a micro-USB 2.0 full-speed interface is used for battery recharge, while a standard Bluetooth technology for short distance data communication is employed by using the *BT33* [29] class 1.5 micro-sized (11.6×13.5 mm${}^{2}$) Bluetooth V3.0 module provided by Amp’ed RF/STMicroelectronics (San Jose, CA, USA).

#### 2.1.5. Environmental Sensors

Besides the MIMU, D-MuSe comes with additional sensing units, making it suitable for a wide range of applications: a temperature sensor which is integrated in the microcontroller with a typical accuracy of about $\pm 1{}^{\circ }$C and a high accuracy pressure sensor *LPS25HB* [33], which ensures a resolution of 0.01 hPa root mean square (RMS).

#### 2.1.6. Memory

In addition to the internal microcontroller flash memory (512 kB) and SRAM (128 kB), a 16 MB flash-NOR non-volatile memory was used to store data for continuous recordings.

#### 2.1.7. PCB Fabrication and Power Supply

**Figure 4:**
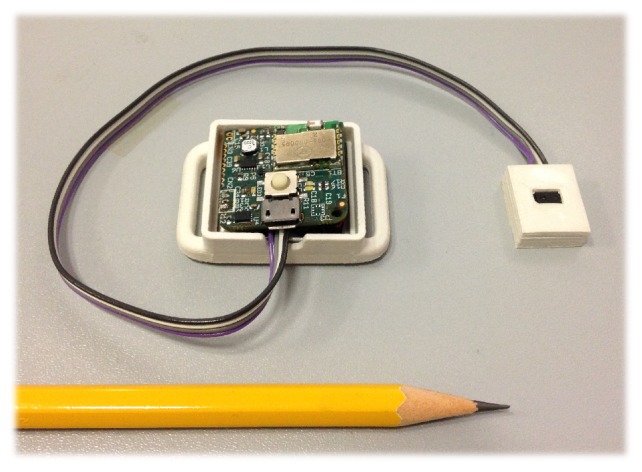
The D-MuSe device including a Li-poly battery.

A 4-layer PCB technology has been adopted to minimize the area occupancy and achieve a form factor of 25×25 mm${}^{2}$. The power can be supplied by either a wired connection or a battery. For this study, a 3.7 V 210 mAh lithium polymer battery is used (Figure 4).

### 2.2. Accuracy of the Distance Estimation

We analyzed the following factors, which could potentially affect the accuracy associated with distance estimation:

(i) Colors of the target surface (red, green, blue, yellow, white and black);
(ii) Distance (from 20 to 200 mm);
(iii) Angle of incidence ($0^{\circ }$, $30^{\circ }$, $-30^{\circ }$, $60^{\circ }$ and $-60^{\circ }$);
(iv) Relative velocity between the sensor and the target.

The influence of factor (i) was tested in static conditions only, factors (ii) and (iii) were tested both in static and dynamic conditions and factor (iv) was tested only in dynamic conditions. These factors were chosen to cover the range of possible configurations occurring during both normal and pathological human gait such as different shoe colors, different internal-external rotation foot angles, different values of the step width and different foot velocities [3,5,32,34,35].

#### 2.2.1. Experimental Setup

**Figure 5:**
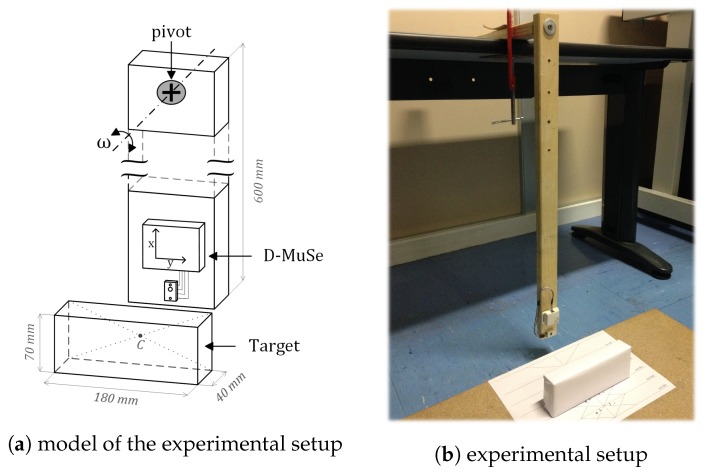
A wooden pendulum with the magneto-inertial measurement unit (MIMU) and the Infrared Time-of-Flight proximity sensor (IR-ToF) attached to its distal end. The stationary target was positioned in front of the pendulum.

The D-MuSe was attached to the end of a wooden pendulum (length 600 mm) simulating the oscillation of a human leg, while a stationary rectangular cuboid target, with dimensions similar to those of a shoe (180×70×40 mm${}^{3}$), was positioned in front of the pendulum (Figure 5). Let *C* be the intersection between the diagonals of the rectangular face facing the sensor, while the distances *d* between the IR-ToF proximity sensor and the target were set using a ruler (gold standard, 1 mm resolution).

#### 2.2.2. Experimental Data Acquisition

**Figure 6:**
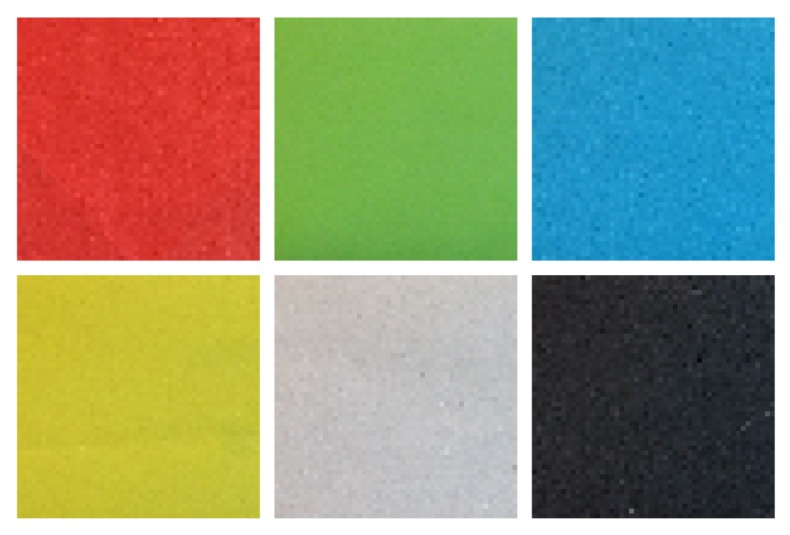
The six colors of the rectangular cuboid targets used during the static acquisitions (red, green, blue, yellow, white and black).

The first part of the experiment consisted of a series of static acquisitions performed by the IR-ToF proximity sensor using six different target colors (red, green, blue, yellow, white and black) varying the distance in the range 0–200 mm with an increment of 20 mm (Figure 6).

Based on the results provided by the preliminary investigation on the influence of the color of the target, we decided to use the white color for the subsequent experimental acquisitions. In static acquisitions, the target was kept stationary in front of the IR-ToF proximity sensor, while during dynamic acquisitions, the pendulum was kept horizontal in the starting position and then released. For both static and dynamic acquisitions, the following experimental conditions were tested:

- AoI $=0^{\circ }$ and d=40, 70, 100, 130, 160, 190 mm;
- AoI $=30^{\circ }$ and d=70, 100, 130, 160, 190 mm;
- AoI $=-30^{\circ }$ and d=70, 100, 130, 160, 190 mm;
- AoI $=60^{\circ }$ and d=100, 130, 160, 190 mm;
- AoI $=-60^{\circ }$ and d=100, 130, 160, 190 mm.

**Figure 7:**
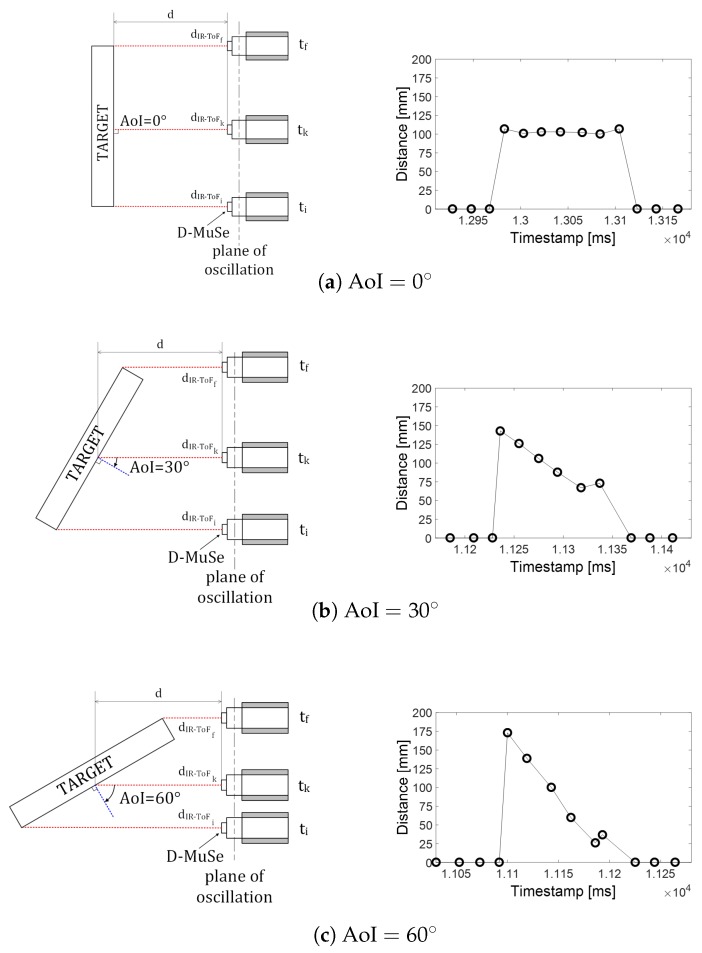
On the left, the top view of the experimental setup for AoI $=0^{\circ }$ (**a**), 30° (**b**) and 60° (**c**). The red dotted line represents the infrared ray emitted by the IR-ToF proximity sensor. *d* is the imposed distance using a ruler, while $d_{IR-ToF_{k}}$ is the distance estimated by the Infrared Time-of-Flight proximity sensor (IR-ToF) when the gyroscope measured a positive/negative peak according to the direction of the pendulum oscillation, while $d_{IR-ToF_{i}}$ and $d_{IR-ToF_{f}}$ are the initial and final estimated distances, respectively. On the right, for each AoI value, an example of the distance values measured by the IR-ToF proximity sensor at *d* = 100 mm is reported. It should be noted that, in dynamic acquisitions, when the AoI differs from zero, the sensor-target distance $d_{IR-ToF_{f}}$ varies with time between $d_{IR-ToF_{i}}$ and $d_{IR-ToF_{f}}$ (Figure 7b,c).

To avoid a collision between the target and the pendulum, for AoI $=\pm 30^{\circ }$ the minimum distance *d* was equal to 70 mm, whereas, for AoI $=\pm 60^{\circ }$, the minimum distance *d* was equal to 100 mm. A schematic representation of the experimental set-up and an example of the raw distance values measured by the IR-ToF proximity sensor for the different AoI values are reported in Figure 7. The distance *d* is measured in correspondence of the lowest point of the pendulum, which is the point with the maximum angular velocity (minimum potential energy and the maximum kinetic energy).

#### 2.2.3. Data Analysis

**Figure 8:**
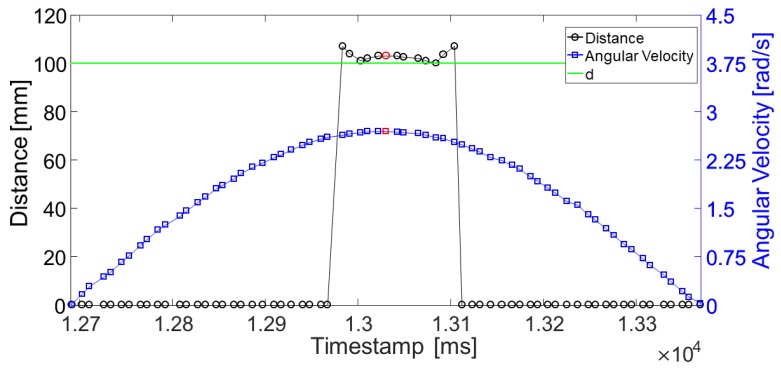
An example of the readings, provided by the Infrared Time-of-Flight proximity sensor (IR-ToF) and re-sampled at 100 Hz, is reported for an oscillation *k* with an AoI equal to $0^{\circ }$ and d=100 mm. The value of $d_{IR-ToF_{k}}$, obtained in correspondence of the angular velocity peak (red square), is reported with a red circle.

Magneto-inertial data were acquired at 100 Hz, while the distance measurements were acquired at 50 Hz (maximum frequency allowed). To provide a continuous estimate of the distance at 100 Hz, the distance measurements were linearly interpolated and then re-sampled at 100 Hz. During static acquisitions, the sensor-target distance was determined by averaging 30 readings. For each dynamic acquisition, we extracted the set of distance values $d_{IR-ToF_{k}}$ provided by the IR-ToF sensor, one for each *k*-th oscillation, while the angular velocity measured in correspondence of the lowest point of the pendulum varied between 1 and 6 rad/s (≈30 oscillations) (Figure 8).

For each dynamic acquisition, defined by specific values of AoI and *d*, the following quantities were computed:

(4) $$e_{AoI,d}=\frac{1}{N}\sum_{k=1}^{N}\left(d_{IR-ToF_{k}}-d\right),$$

(5) $$sd_{AoI,d}=\sqrt{\frac{1}{N-1}\sum_{k=1}^{N}{\left(d_{IR-ToF_{k}}-d\right)}^{2}},$$

(6) $$mae_{AoI,d}=\frac{1}{N}\sum_{k=1}^{N}\left|d_{IR-ToF_{k}}-d\right|,$$

(7) $$mae_{{\%}_{AoI,d}}=\frac{mae_{AoI,d}}{d}\cdot 100,$$

where $d_{IR-ToF_{k}}$ is the distance estimated by the IR-ToF proximity sensor when the gyroscope measured a peak, *d* is the true distance and *N* is the number of oscillations with angular velocity between 1 and 6 rad/s. Furthermore, for each AoI value, the average values of latter indices were computed over the different distances (E, SD, MAE and MAE${}_{\%}$).

### 2.3. Example of Application: Inter-Foot Distance Estimation during Gait

**Figure 9:**
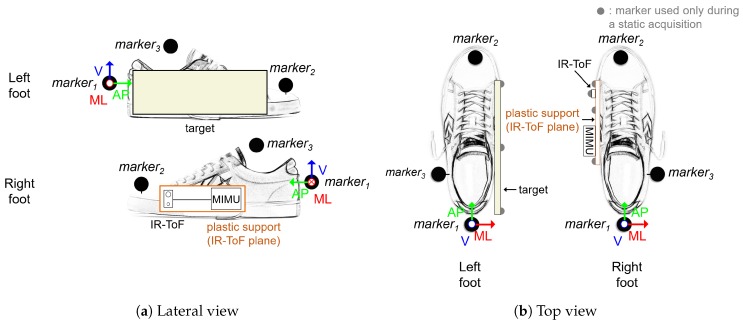
D-MuSe and markers placement on the feet. The origin of the coordinate system was aligned with marker_1_.

In this section, we present an example of the use of the D-MuSe platform for the analysis of human gait. In particular, IR-ToF technology can provide information about the instantaneous or average distance between selected points of the feet (IFD) when they face each other (during mid-swing and mid-stance phases). To this purpose, the D-MuSe platform was attached on a plastic rigid support and positioned on the right foot with the IR-ToF proximity sensor positioned orthogonal to the support and close to the first metatarsophalangeal joint. To avoid measurement uncertainties due to the irregular shape of the shoe, a rectangular target (200×100 mm${}^{2}$) was attached on the medial side of the left foot. A cluster of three markers was placed on each foot to define a coordinate system (Figure 9).

The target and the D-MuSe geometries were acquired and expressed in the relevant coordinate systems from the positions of seven additional markers acquired during a static acquisition (one on the IR-ToF proximity sensor, three on the support and three on the target) (Figure 9b). These markers were removed before acquiring the dynamic trials. Marker positions were recorded using a 10-camera stereo-photogrammetric system (SP) (Vicon Motion Systems, Oxford, UK; 100 Hz). Dynamic experimental data were acquired on a healthy subject during a six-meter straight walk at comfortable speed (0.9 m/s) (three trial repetitions were performed). IFD marker-based reference values were calculated as the distance between the IR-ToF proximity sensor center and the intersection point between the normal to the IR-ToF proximity sensor plane, passing through the IR-ToF proximity sensor center, and the target plane placed on the left foot. For each gait cycle, mean values of the distances provided by IR-ToF and SP during swing and stance phases of the right foot were computed and the absolute differences between IR-ToF proximity sensor and SP mean distance values derived. The overall mean error with standard deviation (E ± SD), mean absolute error (MAE) and mean absolute percentage error (MAE${}_{\%}$) were computed averaging differences over gait cycles and trials.

## 3. Results

### 3.1. Accuracy Evaluation of the Distance Estimation

The performance of the IR-ToF proximity sensor obtained for different colors of the target are reported in Table 1. When the black target was used, the IR-ToF proximity sensor could not measure distances larger than 140 mm.

The accuracy of the distance estimates for an AoI equal to $0^{\circ }$ and d=40, 70, 100, 130, 160, 190 mm using a white rectangular cuboid target are reported, for both static and dynamic acquisitions, in Table 2. In static acquisitions, MAE${}_{\%}$ ranged from 0.8% for d=160 mm to 2.5% for d=40 mm, while, in dynamic acquisitions, the value of MAE${}_{\%}$ ranged from 0.8% for d=160 mm to 2.3% for d=40 mm.

Distance estimation errors for the different AoI values are reported, for both static and dynamic acquisitions, in Table 3. In static acquisitions, E (SD) ranged from 0.2 mm (1.3 mm) for AoI $=0^{\circ }$ to −7.8 mm (1.7 mm) for AoI $=-60^{\circ }$. In dynamic acquisitions, E (SD) ranged from 0.5 mm (1.4 mm) for AoI $=0^{\circ }$ and −9.9 mm (26.9 mm) for AoI $=-60^{\circ }$. In static acquisitions the MAE${}_{\%}$ values varied from 1.4% for AoI $=0^{\circ }$ to 5.0% for AoI $=-60^{\circ }$, whereas, in dynamic acquisitions, they varied from 1.5% for AoI $=0^{\circ }$ to 19.2% for AoI $=-60^{\circ }$.

**Figure 10:**
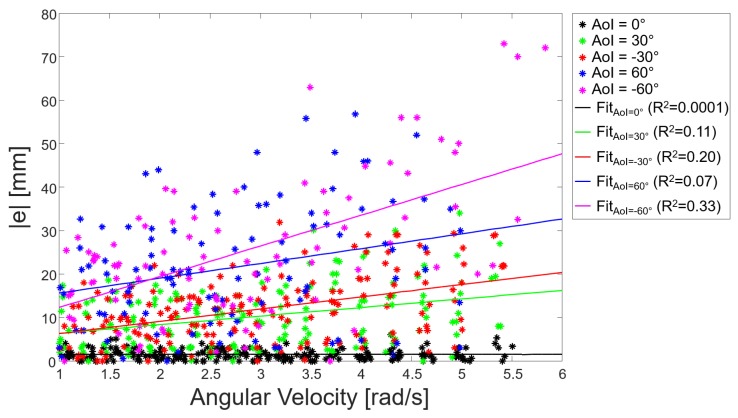
The absolute values of the errors *e* computed by the IR-ToF proximity sensor during all dynamic experiments are reported with a different color for each AoI value. Furthermore, for each AoI value, a colored line showed the trend of the absolute errors with respect to the angular velocity.

In addition, for each AoI value, the relationship between the absolute values of the errors *e* and the angular velocity values during all dynamic acquisitions was investigated by performing a first order polynomial regression (Figure 10).

### 3.2. Feasibility of the Inter-Foot Distance Estimation during Gait

Indicative results from IFD estimation during gait on a single healthy subject are reported in Table 4.

## 4. Discussion

In the present study, we described and tested a wearable platform (D-MuSe), specifically designed for human gait analysis applications, which integrates a MIMU module with an IR-ToF proximity sensor, capable of providing inter-object distance measurements. Thanks to the integration of a millimeter-resolution proximity sensor, D-MuSe may increase the potentiality of traditional magneto-inertial units [9,12] for stability analysis during static and dynamic motor tasks during daily life activities. Since the typical range performances of the IR-ToF sensor (*VL6180X*), reported in the specifications [27], only refer to static conditions and for an AoI equal to zero, the applicability of this technology for human movement analysis applications required further investigations. The present study aimed at filling this gap by performing a thorough testing of the IR-ToF proximity sensor under experimental conditions resembling those encountered when analyzing the feet motion during gait.

A preliminary target color test showed that the IR-ToF technology is not sensitive to the variations of target color except when black is used. In fact, the MAE varied between 1.3 and 2.1 mm among all target colors in the range 20–200 mm (except for black). With a black target, the IR-ToF proximity sensor could only measure distances up to 140 mm and the accuracy of the distance estimation was lower (MAE = 4.1 mm). This finding was in accordance with the study of Lachat et al. [36] performed on a Kinect v2 sensor (Microsoft, Redmond, WA, USA) for close range 3D modelling and it revealed that caution should be paid when dark shoes are used. However, if strictly necessary, errors might be reduced by performing specific black-target calibration of the IR-ToF proximity sensor and by adjusting the measurement range for larger distances (up to 600 mm).

The device performance was tested both in static and dynamic conditions at distances in the range of 40–190 mm and sensor-target orientations between $0^{\circ }$ and $\pm 60^{\circ }$ . The latter values were chosen to include values of step width and foot progression angles, similar to those observable during normal and pathological gait [13]. In general, for AoI equal to $0^{\circ }$, the IR-ToF proximity sensor performed equally well both in static and dynamic acquisitions among all distances, while errors increased as the AoI increased. During static acquisitions, MAE values varied between 1.4 and 7.8 mm when increasing the AoI up to $\pm 60^{\circ }$. The latter trend is likely to occur because the incident rays emitted by the IR illumination cone, striking the non-orthogonal target surface, are reflected with an angle of incidence equal to the target orientation, and this would modify the phase shift once detected by the view cone. In the dynamic acquisitions, MAE values varied between 1.5 mm (AoI $=0^{\circ }$) to 25.6 mm (AoI $=-60^{\circ }$). The larger errors, observed during the dynamic acquisitions compare to the static acquisitions, may be related to the unavoidable uncertainty associated to the correct identification of the instant of time during which the IR-ToF proximity sensor is in front of the target (imposed known distance) (Figure 5). In fact, when the AoI is equal to $0^{\circ }$ (pendulum oscillating parallel to the target surface), the sensor-target distance is constant (Figure 7a); conversely, when AoI is different from $0^{\circ }$, the sensor-target distance changes with time (Figure 7b,c). Since the gyroscope *ODR* is 100 Hz, the time difference between two consecutive samples is 10 ms. This implies that the maximum error in the identification of the instant of time, which maximizes the angular velocity, is 5 ms. By performing simple geometrical calculation, given AoI $=\pm 60^{\circ }$, a length of the pendulum l=600 mm and ω=6 rad/s, a time shift of 5 ms causes an error in the estimated distance equal to ±31 mm. This hypothesis was preliminarily confirmed by the experimental results reported in Figure 10, which showed a weak but positive correlation between the error magnitude and the angular velocity for AoI different from zero.

As we mentioned, the large majority of the studies using inertial and distance data has not quantified the level of accuracy of the distance estimates in static and dynamic conditions. A preliminary evaluation of an IR-LI sensor (*GP2Y0A41SK0F*, Sharp Corporation, Osaka, Japan) performance can be found in Trojaniello et al. [18]. When comparing the errors found in static conditions in the present study with those reported in Trojaniello et al., an error reduction from 5.5 mm (4%) to 1.4 mm (1.4%) was observed (AoI $=0^{\circ }$). Similarly, in dynamic conditions (pendulum versus leg swinging), errors were reduced from 2.7 mm (5%) to 1.5 mm (1.5%).

The small errors in the IFD estimation (≅5 mm), found in the preliminary experiment during gait, confirmed the potential of the D-MuSe platform for gait analysis applications. However, given the limitations found in the pendulum experiments (worsening of the performance for AoI values larger than $30^{\circ }$, high pendulum angular velocity and black target color), further investigations on normal and pathological gait are necessary. In particular, future studies should pay attention to the effects of different gait speeds, gait patterns, excessive foot progression angles and different types of shoes.

## 5. Conclusions

The D-MuSe platform, presented and tested in this work, is based on the combination of a wearable MIMU connected to an IR-ToF proximity sensor. This system may be highly valuable for the measurement of short range distances. The main aim of this paper was the validation of the IR-ToF proximity sensor in static and dynamic conditions similar to those encountered during various human movements. The results showed that the IR-ToF proximity sensor is not sensitive either to variations in the distance or in the target color (except for black), but it is sensitive to variations of the angle of incidence. D-MuSe allowed for estimating distances up to 200 mm with a satisfactory accuracy for an AoI up to $\pm 30^{\circ }$ in both static (MAE${}_{\%}=2.5\%$) and in dynamic conditions (MAE${}_{\%}$ up to 10.2%). We demonstrated that for AoI equal to $\pm 60^{\circ }$ (i.e., subjects affected by bone deformities or excessive external feet rotations), the quality of the distance estimation substantially decreases with an MAE${}_{\%}$ up to 5% and 19.2% in static and dynamic conditions, respectively. Despite the very preliminary stage of the example of application, D-MuSe showed promising results for measuring the IFD during gait (MAE = 5.0). In the future, the application of the present methodology on a larger number of human subjects and the development of new algorithms, fusing IR-ToF distance estimates and magneto-inertial data (accelerations, angular velocities and local magnetic field intensity), will be assessed in both static (e.g., balance tests) and dynamic motor tasks (e.g., gait analysis).

## Figures and Tables

**Table 1:** Overall mean error with standard deviation (E (SD)), mean absolute error (MAE) and mean absolute percentage error (MAE${}_{\%})$ of the distance for the six target colors.

Color	E (SD) [mm]	MAE [mm]	MAE${}_{\%}$ [%]
Red	1.1 (1.2)	1.3	3.0
Green	1.5 (1.1)	1.5	3.7
Blue	2.1 (1.2)	2.1	4.2
Yellow	1.1 (1.1)	1.5	3.9
White	1.6 (1.1)	1.6	3.8
Black ^1^	14.1 (1.2)	4.1	7.2

${}^{1}$ The distance range was 20–140 mm because no distance values were obtained for d=160, 180 and 200 mm.

**Table 2:** Mean error with standard deviation (e (sd)), mean absolute error (mae) and mean absolute percentage error (mae${}_{\%})$ for AoI = $0^{\circ }$ during both static and dynamic acquisitions using a white rectangular cuboid target.

	Static			Dynamic		
d [mm]	e (sd) [mm]	mae [mm]	mae${}_{\%}$ [%]	e (sd) [mm]	mae [mm]	mae${}_{\%}$ [%]
40	0.7 (1.0)	1.0	2.5	0.5 (1.1)	0.9	2.3
70	0.2 (1.4)	1.0	1.5	0.7 (1.5)	1.3	1.8
100	0.6 (1.5)	1.1	1.1	0.7 (1.5)	1.3	1.3
130	1.6 (1.5)	1.8	1.4	1.9 (1.5)	2.0	1.5
160	0.5 (1.5)	1.2	0.8	1.1 (1.2)	1.3	0.8
190	−2.5 (1.2)	2.5	1.3	−2.2 (1.7)	2.3	1.2

**Table 3:** Overall mean error with standard deviation (E (SD)), mean absolute error (MAE) and mean absolute percentage error (MAE${}_{\%})$ of the distance for the five conditions using a white target.

Condition		E (SD) [mm]	MAE [mm]	MAE${}_{\%}$ [%]
AoI = 0${}^{\circ }$	Static	0.2 (1.3)	1.4	1.4
	Dynamic	0.5 (1.4)	1.5	1.5
AoI = 30${}^{\circ }$	Static	2.4 (1.5)	2.7	2.5
	Dynamic	3.1 (11.0)	9.8	9.5
AoI = −30${}^{\circ }$	Static	−3.4 (1.5)	3.6	2.5
	Dynamic	−5.6 (12.4)	11.9	10.2
AoI = 60${}^{\circ }$	Static	0.4 (1.9)	3.6	2.5
	Dynamic	−8.0 (24.2)	22.8	16.3
AoI = −60${}^{\circ }$	Static	−7.8 (1.7)	7.8	5.0
	Dynamic	−9.9 (26.9)	25.6	19.2

**Table 4:** Average of the inter-foot distance values with standard deviation ($\bar{IFD}$ (SD)), overall error with standard deviation (E (SD)), mean absolute error (MAE) and mean absolute percentage error (MAE${}_{\%}$) during gait.

$\bar{\mathrm{IFD}}$ (SD) [mm]	E (SD) [mm]	MAE [mm]	MAE${}_{\%}$ [%]
83.6 (11.0)	3.0 (7.2)	5.0	5.7

## Abbreviations

The following abbreviations are used in this manuscript:

IMU	Inertial Measurement Unit
US	Ultrasounds
IR-LI	Light Intensity Infrared
VC	Video Camera
ODR	Output Data Rate
IR-ToF	Infrared Time-of-Flight
AoI	Angle of Incidence
IFD	Inter-Foot Distance
D-MuSe	Distance-MultiSensing
MIMU	Magnetic and Inertial Measurement Unit
